# Development of a shear stress-free microfluidic gradient generator capable of quantitatively analyzing single-cell morphology

**DOI:** 10.1007/s10544-017-0222-z

**Published:** 2017-09-07

**Authors:** David Barata, Giulia Spennati, Cristina Correia, Nelson Ribeiro, Björn Harink, Clemens van Blitterswijk, Pamela Habibovic, Sabine van Rijt

**Affiliations:** 1 Department of Tissue Regeneration, MIRA Institute for Biomedical Technology and Technical Medicine, University of Twente, P.O. Box 217, 7500 AE Enschede, The Netherlands; 2 Department of Instructive Biomaterials Engineering, MERLN Institute for Technology-Inspired Regenerative Medicine, Maastricht University, P.O. Box 616, 6200 MD Maastricht, The Netherlands; 3 Instituto de Engenharia Mecânica, Laboratório Associado de Energia, Transportes e Aeronáutica, Instituto Superior Técnico, Universidade de Lisboa, Av. Rovisco Pais, 1049-001 Lisbon, Portugal; 4 Department of Complex Tissue Regeneration, MERLN Institute for Technology-Inspired Regenerative Medicine, Maastricht University, P.O. Box 616, 6200 MD Maastricht, The Netherlands

**Keywords:** Microfluidics, Image analysis, Concentration gradient, Cytochalasin D

## Abstract

Microfluidics, the science of engineering fluid streams at the micrometer scale, offers unique tools for creating and controlling gradients of soluble compounds. Gradient generation can be used to recreate complex physiological microenvironments, but is also useful for screening purposes. For example, in a single experiment, adherent cells can be exposed to a range of concentrations of the compound of interest, enabling high-content analysis of cell behaviour and enhancing throughput. In this study, we present the development of a microfluidic screening platform where, by means of diffusion, gradients of soluble compounds can be generated and sustained. This platform enables the culture of adherent cells under shear stress-free conditions, and their exposure to a soluble compound in a concentration gradient-wise manner. The platform consists of five serial cell culture chambers, all coupled to two lateral fluid supply channels that are used for gradient generation through a source-sink mechanism. Furthermore, an additional inlet and outlet are used for cell seeding inside the chambers. Finite element modeling was used for the optimization of the design of the platform and for validation of the dynamics of gradient generation. Then, as a proof-of-concept, human osteosarcoma MG-63 cells were cultured inside the platform and exposed to a gradient of Cytochalasin D, an actin polymerization inhibitor. This set-up allowed us to analyze cell morphological changes over time, including cell area and eccentricity measurements, as a function of Cytochalasin D concentration by using fluorescence image-based cytometry.

**Electronic supplementary material:**

The online version of this article (10.1007/s10544-017-0222-z) contains supplementary material, which is available to authorized users.

## Introduction

In the past two decades, high-throughput screening (HTS) and high-content screening (HCS) have become major landmarks in the field of drug discovery, leading to fast identification of new therapeutic molecules and novel genetic engineering strategies (Zhao et al. 2015; Lovitt et al. 2013; Carlson-Stevermer et al. 2016; Macchi et al. 2016). This has largely been accomplished by miniaturization and automation, for example by developing large multiwell plate-based screens (Nishihara et al. 2016; Vrij et al. 2016; Spencer et al. 2016), customized biomolecule/cell arrays (Beachley et al. 2015; Zhao et al. 2015; Kwon et al. 2011), cell sorting (Liu et al. 2016; Stowe et al. 2015; Chuang et al. 2014) and microfluidics (Du et al. 2016; Barata et al. 2016). Microfluidics has made an important contribution to HTS and HCS methodologies by enabling experiments with small amounts of reagents and low cell numbers. This is especially useful for the development of biological screens for cells with limited availability (e.g. primary (pluripotent) cells) and in addition, considerably reduces the costs of automation.

Microfluidic systems are capable of manipulating small volumes of fluids in a controlled manner, which enables the integration of multiple parallel, combinatorial or sequential processing steps (Harink et al. 2013; Du et al. 2016; Kim et al. 2015; Santoso et al. 2015; Barata et al. 2016). In particular, by closely controlling fluid flows, microfluidic devices can be used to generate gradients of, for example, soluble molecules. This capability can be exploited to expose cultured cells to a large range of concentrations of the compounds of interest in a single experiment (Harink et al. 2015; Kilinc et al. 2016; Xiao et al. 2014; Zou et al. 2015). The main mechanisms to create gradients using microfluidics involve the use of parallel laminar flows or the establishment of diffusion through a source-sink system. The type of mechanism determines the profile of the gradient and its hydrodynamic characteristics inside the device (Berthier and Beebe 2014; Kim et al. 2010).

While the microfluidic technology possesses an enormous potential to generate a multitude of conditions within a single experiment, the throughput and the content of screening in microfluidic devices is still largely dependent on the capabilities of the assay used to measure the biological response. Recent efforts have been invested in image-based cytometry methods that are suitable for single-cell analysis. An advantage of such a method is that it allows multiparametric biochemical probing of individual cells within a population, rather than the population as a whole (Lowes et al. 2011; Schramm et al. 2011; Ito et al. 2014). For microfluidic devices employing 2D cell culture, image cytometry has proven to be a simple and efficient method with multiplex capabilities and therefore a high throughput of screening. Application examples include studies on cell morphology, viability, cell cycle and protein expression (Kamei et al. 2010; Ye et al. 2007; Harink et al. 2015; Yoo et al. 2013). For example, Yoo et al. (Yoo et al. 2013) described a microfluidic image cytometry device capable of gradient generation through serial dilutions running in parallel chambers, where the DNA content (nuclei size and propidium iodide staining intensity) was used for the assessment of cell cycle in response to a toxin, paclitaxel. In our earlier study (Harink et al. 2015), we used a diffusion-based microfluidic gradient platform to study the nuclear translocation of hypoxia-inducible factor 1 (HIF1) as a concentration-dependent response to hypoxia-mimicking molecule phenantroline.

In the current work, we developed a microfluidic platform that can be used to study the effects of a gradient of soluble compounds on adherent cells. This platform consists of a microfluidic network with five serially connected cell culture chambers, in which a gradient of soluble compounds can be generated by diffusion. The design of the cell culture chambers and the fluidic network was modelled and fabricated with the aim to provide a wide range of concentrations with a linear gradient profile, while minimizing the shear stress inside the cell culture chambers. Furthermore, the cell culture area per chamber allowed the culture of hundreds of cells, in order to increase the sample size for image-based analysis of cell behavior. To validate the microfluidic set up, MG-63 osteosarcoma cells were cultured inside the cell culture chambers and exposed to a gradient of Cytochalasin D, a potent actin polymerization inhibitor (Schliwa 1982; Scherlach et al. 2010). Morphological changes in time, in terms of cell area and eccentricity were measured using whole cell fluorescence live staining for image-based cytometry. Cytochalasin D has received interest for its therapeutical potential, e.g. in tumour treatment (Trendowski 2015; Trendowski et al. 2015; Huang et al. 2012), as an adjuvant of antibiotics (Dey et al. 2015) and in regenerative medicine (Kim et al. 2012; Pirttiniemi and Kantomaa 1998). The ability to study the effects of gradients of molecules in solution at the cellular level in a high-throughput fashion will aid our understanding of complex molecular pathways such as those involved in cytoskeleton dynamics.

## Materials and methods

### Chemicals and reagents

Silicon wafers (p-type, single side polished, Ø =100 mm) were purchased from Silicon Materials. Positive tone photoresist and developer were purchased from AZ Electronic Materials. Piranha solution (H_2_O_2_:H_2_SO_4_, 1:3) was produced by mixing 1 part of sulfuric acid, from Sigma-Aldrich, with 3 parts of hydrogen peroxide, from Honeywell. SU-8 photoresist and developer were purchased from Microchem and polydimethylsiloxane (PDMS) Sylgard 184 elastomer kit from Dow Corning. Phosphate buffered saline (PBS), Minimum Essential Medium a (a-MEM), sodium pyruvate and 0.25% trypsin in ethylene-diamine-tetraacetic acid (EDTA) were obtained from Gibco, Life Technologies. Fetal bovine serum (FBS) was purchased from Lonza and CellTracker™ Green CMFDA dye from Molecular Probes, Life Technologies. N-(2-Hydroxyethyl)piperazine-N′-(2-ethanesulfonic acid (HEPES)**,** Cytochalasin D and CF™ 568 maleimide were purchased from Sigma-Aldrich. Human osteosarcoma cell line MG-63 (ATCC® CRL1427™) was obtained from ATCC-LGC.

### Device fabrication

The master mold was produced by a dual-step soft-lithography process. In the first step, to produce lower structures comprising the 37.5 μm-high cell culture chamber and channels designed to create diffusion inside the cell culture chamber, a silicon wafer was cleaned and masked by a positive-tone resist through a photolithography process, prior to processing by deep reactive ion etching (Adixen SE, Alcatel). Subsequently, resist was removed and substrate was cleaned in piranha solution. In the second step, to produce the siding channel for cell culture medium perfusion with the height of 150 μm, a layer of SU-8 was added on top of the structures produced in the first step, and patterned using a photolithographic process.

To create the PDMS microfluidic network, a 10:1 ratio mixture between the elastomer and the curing agent was prepared. After filling the mold inside a reservoir with PDMS, the mixture was degassed in a vacuum chamber. Curing was performed in an oven at 80 °C for 1 h. The devices were then cut by a razor blade and the fluidic connection ports were punched. To assemble the device, the PDMS microfluidic network was irreversibly bonded to the glass substrate after both surfaces were treated with air plasma for 60 s (Plasma cleaner/sterilizer Harrick®, High RF level, 0.4 mbar).

**Fig. 1:**
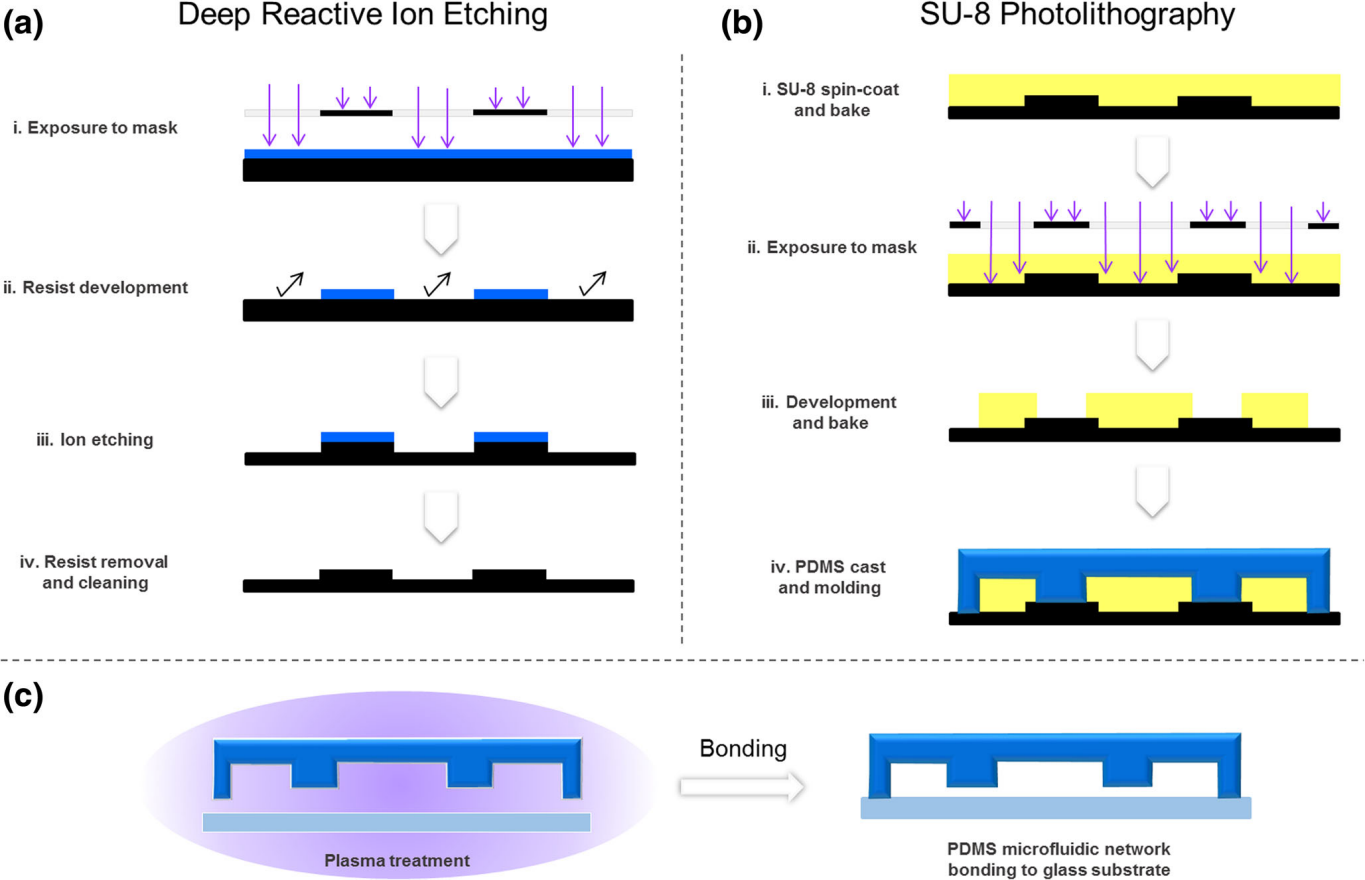
Simplified multistep process flow for fabrication of the microfluidic device. **a** Photolitography of positive tone resist followed by deep reactive ion etching and cleaning. **b** Microstructuring of SU-8 layer by photo-litography on top of previously microfabricated silicon etched layer, followed by molding of PDMS microfluidic network. **c** Bonding of the dual-height PDMS microfluidic network component to a glass substrate

The device fabrication flow is illustrated in Fig. 1.

### Velocity field and concentration profiles simulation

A 3D model simulation was built by Finite Element Modelling (FEM) based on three major compartments of the device: (1) two independent side-channels perfused in parallel, fed at same speed and with equilibrated pressure levels, connected to (2) the culture chamber, through (3) an array of smaller channels at each side (model geometry shown in Supplementary Fig. 1). This model simulates a microreactor, within which unbalanced species concentrations generate a continuous diffusion-based gradient. The model simulations were defined using the Transport of Diluted Species interface (describing Fick’s law) for diffusion phenomena calculations and, coupled to Laminar Flow interface (governed by Navier-Stokes equations, Re < 1, neglected inertia, incompressible flow), for fluid dynamics. Both simulations were performed in COMSOL Multiphysics 4.3 software (Harink et al. 2015; Bruus 2015).

### Cell culture

Human osteosarcoma MG-63 cells were cultured in alpha minimum essential medium (α-MEM) supplemented with 10% fetal bovine serum (FBS), 1% penicilin-streptomycin, 1% glutamine, 1% HEPES and 1% sodium pyruvate. The cells were cultured at 37 °C in a humidified atmosphere of 5% CO_2_. Medium was replaced every 2 to 3 days. Cells were sub-cultured in standard culture flasks upon reaching 80–90% confluence, and then trypsinized with 0.25% trypsin/EDTA for 5 min at 37 °C.

One day before seeding in the microfluidic device, the cells were fluorescently labeled using CellTracker™ Green CMFDA dye (10 μM in complete cell culture medium) by incubation at 37 °C for 45 min in a tissue culture flask. After incubation, the medium was refreshed, and cells were kept in culture until further use.

### Microplate cell culture model for Cytochalasin D dose-response assessment

Cells were seeded in 96-well microplate wells at a density of 5000 cells / well and cultured for 24 h. A second 96-well microplate was prepared with dilution series of Cytochalasin D for readily dispensing into the cell culture microplate after the 24 h. Directly after exposure of the cells to Cytochalasin D, cells were imaged in BD Pathway™ 435 inverted fluorescent microscope, in a 2 × 2 montage at a magnification of 10×, through a time series at 2, 15, 30, 60 and 90 min.

### Microfluidic setup

CellTracker™ Green CMFDA-labeled MG-63 cells were seeded from a suspension of 5 × 10^5^ cell/ml into the microfluidic device and cultured for 24 h prior to the assay. A 250 μl glass syringe (SGE) containing a fluorescent red dye, CF™ 568 maleimide (1:10,000 *v*/v) for the characterization of the gradient formation, and Cytochalasin D, diluted in the cell culture medium to a final concentration of 5 μg/ml was prepared. A second glass syringe contained only the cell culture medium. A syringe pump (Nexus 3000, Chemyx) was loaded with the two syringes, both connected by tubing to the microfluidic device, and assembled into the BD Pathway™ 435 inverted fluorescence microscope to allow imaging. The flow was set at 25 μl/h until the dye reached the cell culture chambers, and then reduced to 8 μl/h to establish and maintain the gradient.

### Image acquisition and post-processing

**Fig. 2:**
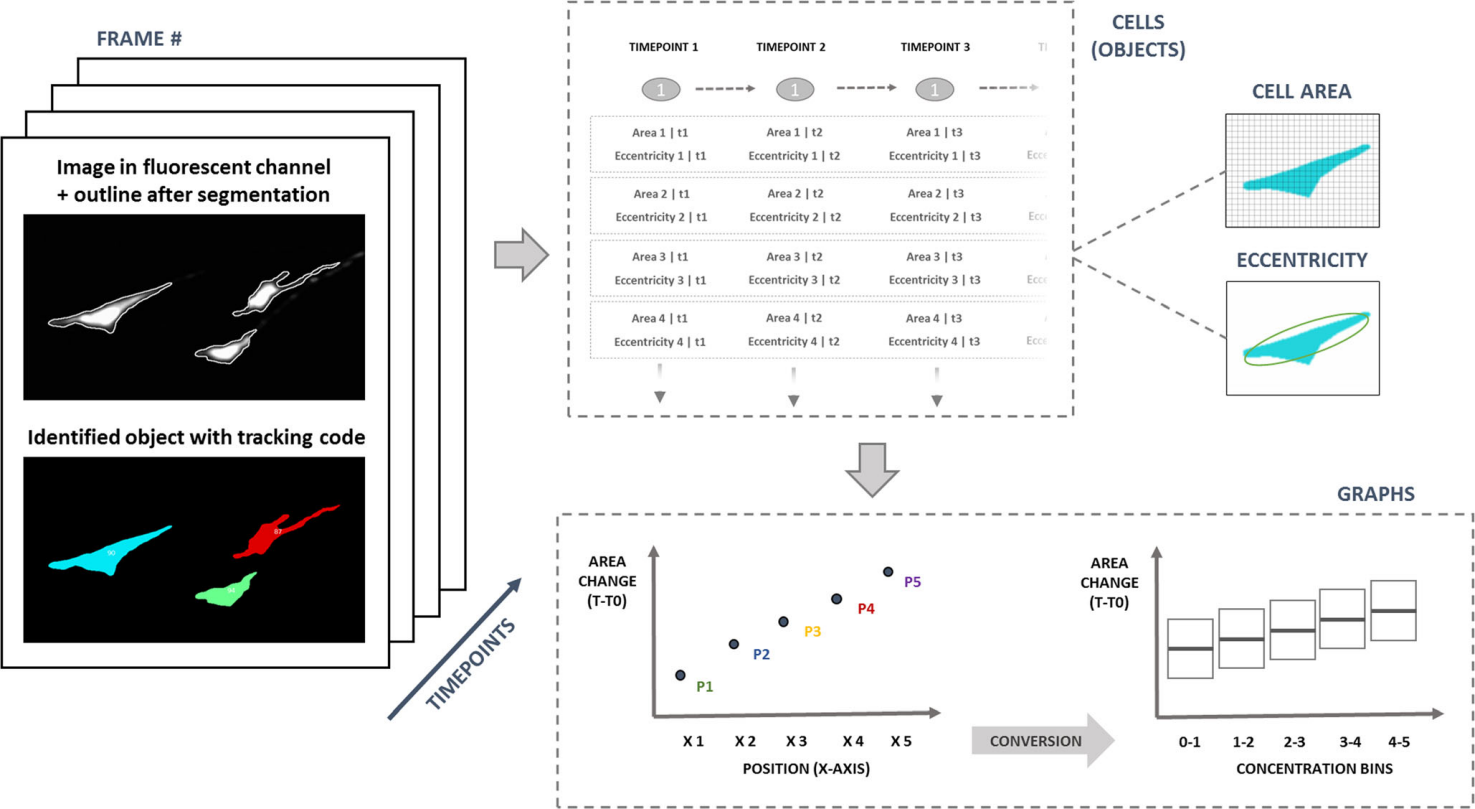
Image analysis workflow including: segmentation (identification of objects, measurements and tracking) in multiple frames from different microfluidic chambers at different time points; assembly of data in tables including measurements for cells at different time points and gradient intensity profiles; and final merge of the data into graphs representing cell morphology measurements as a function of Cytochalasin D concentration

Image acquisition was performed at a magnification of 20×. Multi-frame XY-stitched 7 × 11 images were acquired by alternating between green (CellTracker™ Green CMFDA stained cells) and red (CF™ 568 maleimide dye) channels, in order to image the cells and the Cytochalasin D gradient they were exposed to. Time-lapse cycles included acquisition at the time points of 5, 30, 45 and 60 min. Acquired images were aligned (i.e. image registration) in Image J (FIJI) (Schindelin et al. 2012) for faster computing and better performance in object tracking. The five independent cell culture chambers were analyzed individually at every time point. Quantification of the intensity of CF™ 568 maleimide dye was used as an indirect measurement for the concentration of Cytochalasin D in the cell culture chambers. The dye intensity was plotted as a function of the location inside the cell culture chamber. Cells stained with CellTracker™ Green CMFDA were used for quantitative analysis of the cell morphology in time, in terms of cell area (measured by the number of pixels occupied) and eccentricity as a descriptor of cell shape (with a circle having an eccentricity of 0, and an ellipse which is not a circle, having an eccentricity between 0 and 1). Segmentation and single-cell morphological measurements were performed by combining Image J for image processing, Cell Profiler for segmentation and cell tracking (Carpenter et al. 2006) and Matlab® 2016b (Mathworks) for data analysis (grouping, matching and plotting the quantitative data generated from each image dataset). A schematic of this data analysis process is shown in Fig. 2.

The quantitative results, showing cell area and eccentricity relative differences between time 0 and the time of the measurement are presented as scatter plots. Positive differences in cell area represent a decrease from the initial value, whereas positive differences in eccentricity represent a change in morphology from ellipsoid to a rounder shape.

## Results and discussion

### Finite element modeling and gradient validation

FEM was performed to optimise the design and the dimensions of the various compartments of the device, including the shape and the size of the cell culture chambers and the fluidic network. The final design consisted of large (base area of 3.78 × 1.65–1.32 mm^2^, height of 37.5 μm) hexagonal cell culture chambers, connected on two opposite sides to a perfusion channel (width of 200 μm and height of 150 μm) through an array of small channels (length of 112.5 μm, width of 25 μm, and height of 37.5 μm). While inside the larger perfused channels the laminar flow is expected to be the dominating fluidic regime, the smaller channels, along with the balanced pressure, ensured that inside the cell culture chamber, only diffusion occurs for mass transfer, as is also described for other gradient-based microfluidic systems (Ayuso et al. 2015; Harink et al. 2015).

**Fig. 3:**
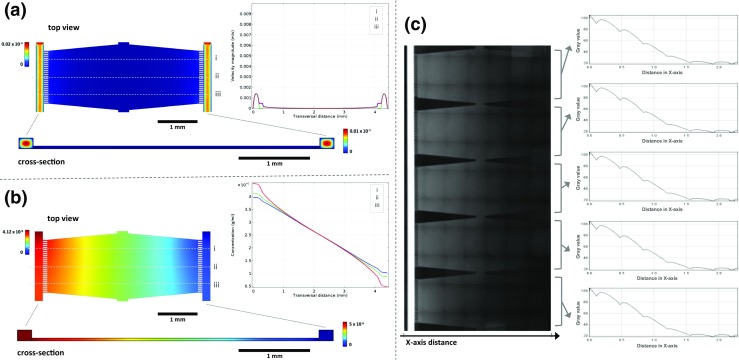
Finite Element Modelling of flow regimes and concentration at steady state in a unit of the microfluidic device. Modeled data obtained by FEM of (**a**) velocity field and (**b**) concentration in the supply side channels, central culture chamber and the connecting small channels, with the corresponding 2D representations (cross section﻿)﻿ and line graphs (representing the three profile lines i, ii, iii). **c** Fluorescence images montage for characterization of CF™ 568 maleimide dye distribution and graphs showing the mean concentration profiles for each chamber

A flow simulation was generated by FEM for a cell culture chamber unit (Supplementary Fig. 1) to predict the concentration gradient profile of a soluble compound formed by diffusion between the “source” and the “sink” channel. The perfusion rate of an aqueous solution through the two side channels was set at 8 μl/h, and equal pressure was assumed for both side channels. The decoupling of the flow regimes between side channels and the cell culture chamber was predominantly achieved by the difference in height between the two components, and the balanced pressures. Therefore, the maximum velocity field was reached in the center of the side channels, becoming lower at the edges (as expected in a laminar flow regime) (Bruus 2015), and being negligible inside the cell culture chamber. Furthermore, the liquid continuity between the side channels and the culture chamber was ensured by small microchannels, which act as resistors, preventing the shear stress to be transmitted to the cell culture chamber (Fig. 3a). Nevertheless, under these conditions, the diffusion of soluble species through the cell culture chamber still occurs, creating a consistent and linear gradient profile (Fig. 3b). The diffusion coefficient was not expected to have an influence on the final steady state of the gradient profile (Harink et al. 2015), but only to affect the time required to reach the equilibrium.

For system validation, a CF™ 568 maleimide solution was added to the cell culture medium in one of the glass syringes while loading the second syringe with medium without the dye. The two syringes were connected to the same high-precision syringe pump to feed the channels, creating an unbalanced source-sink system. As is illustrated in Fig. 3c, it was possible to create a concentration gradient between 0 and 5 μg/ml of the dye inside the 5 cell culture chambers of the device, through the two side channels. The gradient profile exhibited a quasi-linear gradient slope ranging from 100% at the source to 0% at the sink channel. In the Supplementary Fig. 2, representative concentration profiles as a function of the location in the chamber are given for each of the 5 chambers in time. The compound used in this model requires approximately 30 min to reach a steady-state. It should be noted that the gradient profiles were not fully identical in all five chambers; nevertheless, this experiment demonstrated the possibility of creating a broad gradient of concentrations in multiple chambers simultaneously.

The simulations and the initial validation experiments with the fluorescent dye demonstrated that it was possible to create a linear concentration profile in multiple cell culture chambers and, by maintaining the flow rate inside the side channels at 8 μl/h.

### Cytometric validation of cell response to Cytochalasin D

Next, we aimed to establish an image-based method to investigate the concentration-dependent response of cells inside the microfluidic device; and such, by creating a gradient of concentrations of Cytochalasin D. This molecule was previously shown to permeate cell membranes and directly affect cell mobility, adhesion and morphology (Schliwa 1982; Scherlach et al. 2010), making it a useful model drug in mechanistic studies focusing on cytoskeletal transduction (Mak et al. 2016; Martin et al. 2009; Schulze et al. 2009; Schliwa 1982). In the current study, Cytochalasin D was selected for its rapid visible effect on cell morphology, allowing visualization of the concentration-dependent effect on cells by cell shape tracking as a simple imaging readout.

**Fig. 4:**
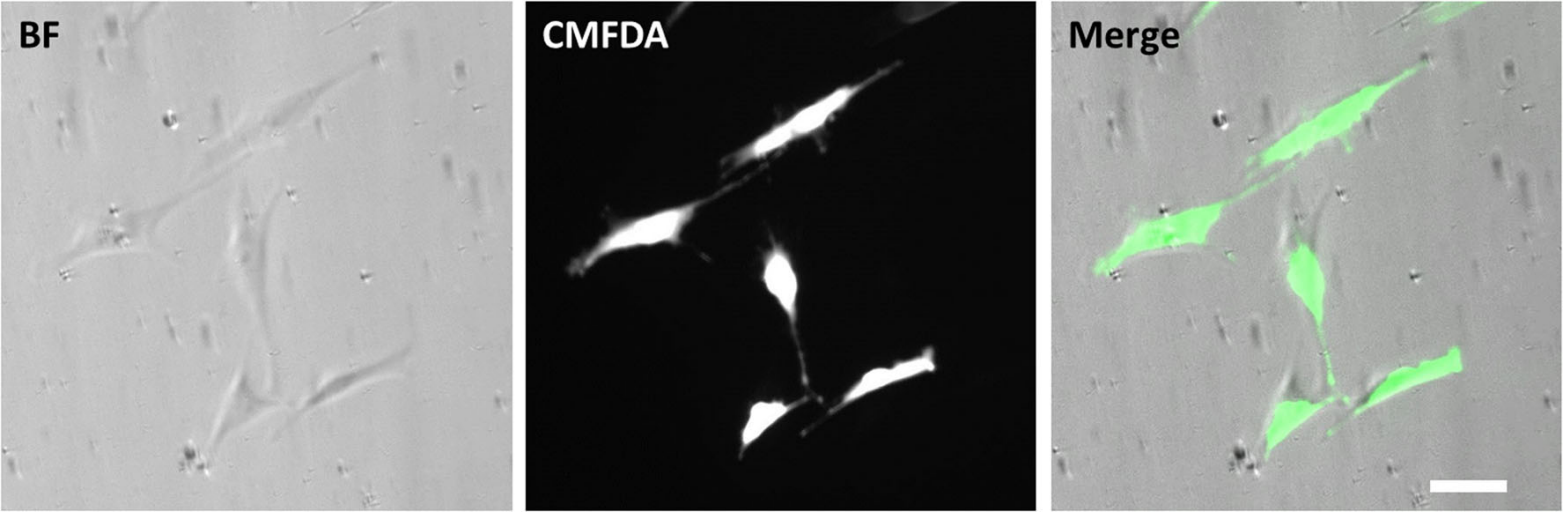
Cell morphology of MG-63 cells in brightfield (BF), in live staining from CellTracker™ Green CMFDA and merged image. Scalebar = 20 μm

To enable the analysis of cell shape changes over time, MG-63 cells were labeled with CellTracker™ Green CMFDA, which stained the cytoplasm with high signal-to-noise ratio (Fig. 4). This dye is easily internalized, passing through the cell membrane into the cytoplasm, where it is transformed into cell membrane-impairment reaction products. The dye is also transferred to daughter cells and allows whole cells to be fluorescent for at least 72 h (ThermoFisher Scientific 2014). Labeling the cells enabled the detection of the morphological changes in cells, which was verified by merging the fluorescent images with images taken in the brightfield mode (Fig. 4). Preliminary experiments in microplate demonstrated a microscopically visible change in cell area of MG-63 cells within 5 min after the exposure to low concentrations of Cytochalasin D (< 5 μg/ml), making the model suitable for the proof-of-concept experiments in the microfluidic device (Supplementary Fig. 3). The rapidly observed shape changes indicate a quick cytoskeletal effect induced by Cytochalasin D. This behavior was already observed at the earliest time point of 2 min, and became slightly more pronounced thereafter.

### Effect of Cytochalasin D gradient on cultured cells in the microfluidic device

For the experiments in the microfluidic device, a concentration range between 0 and 5 μg/ml was selected, based on earlier studies in which the concentrations of up to 5 μg/ml were used to analyze the effect of the drug on cell morphology (Wenzel et al. 2011; Mortensen and Larsson 2003; Schliwa 1982; Schulze et al. 2009; Woods et al. 2008; Rotsch and Radmacher 2000; Wakatsuki et al. 2001). A gradient of Cytochalasin D was created in the five cell culture chambers, showing a profile that was in accordance with the FEM simulation and validation experiments at equilibrium. Oxygen was supplied to the cells through PDMS, which is known to be gas-permeable (Saito et al. 2006; Berthier et al. 2012).

**Fig. 5:**
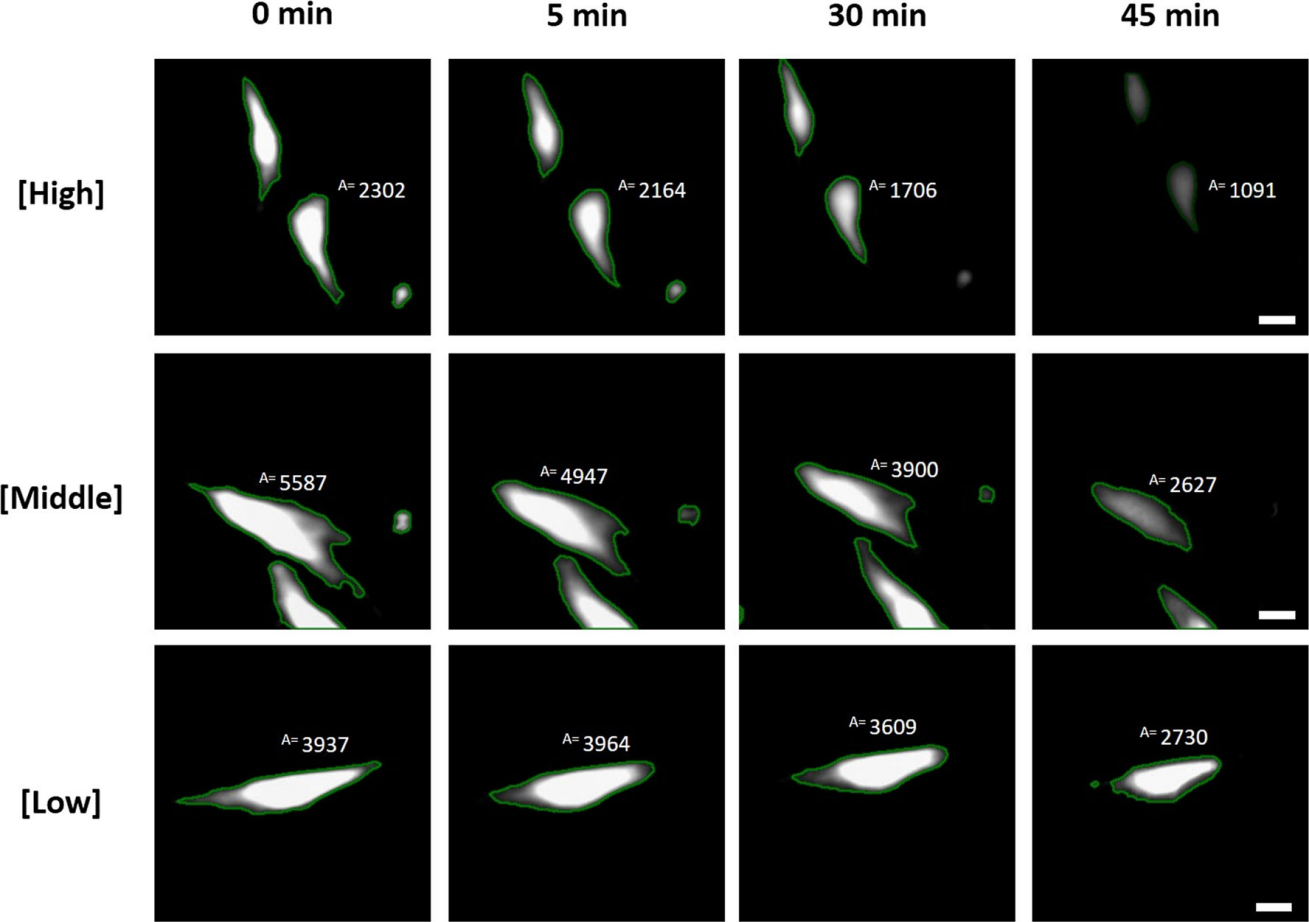
Fluorescence microscopy images of individual MG-63 cells in time in different locations inside the cell culture chamber, representing exposure to different concentrations of Cytochalasin D. The cells were stained with CellTracker™ Green CMFDA and the cytoskeleton is delineated after segmentation. Area (“A”) values are shown in arbitrary units for the largest cell in the frame. Scalebar = 20 μm

As a result of exposure to a gradient of Cytochalasin inside the microfluidic device, a concentration- and time-dependent effect on the area of individual cells could be followed (Fig. 5). A decrease in cell area was observed at higher concentrations of Cytochalasin D, suggesting that a threshold concentration exists at which a biological effect is observed.

The set-up allows for each individual cell within each chamber to be used as individual measurements (Fig. 5). In addition, all cells within each chamber can also be pooled to obtain a large number of data points. Furthermore, the results from each of the five chambers can also be pooled together after correcting for the slightly different drug concentration profiles that were observed between the chambers. Specifically, as was shown in the modeling and validation experiments, and later confirmed with the fluorescent dye experiments, approximately 30 min (or more in the case of pressure instability) was required for the gradients to reach the equilibrium (Supplementary Fig. 2). To correct for the different drug concentration profiles, cell tracking was used to assign concentration values to each cell, and this subsequently allowed for the calculation of the mean concentration Cytochalasin D experienced per cell.

**Fig. 6:**
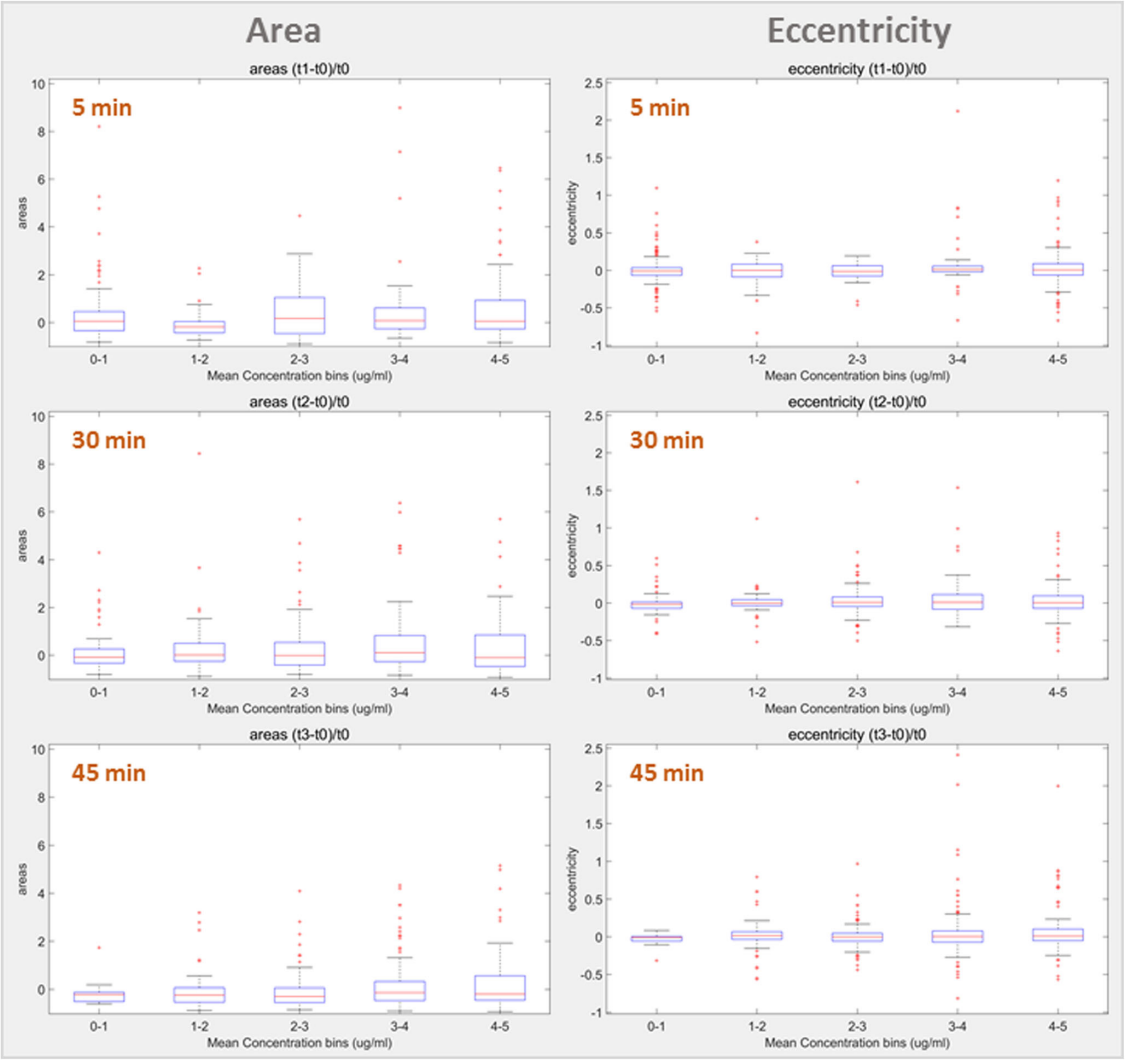
Box plot showing differences in cell area and cell eccentricity of MG-63 cells, normalized for the values at time 0, upon exposure to different concentrations of Cytochalasin D. The compound concentration used is the mean concentration the cells were exposed to between time 0 and the time of the measurement

To show the pooled data from the five cell culture chambers, the results were expressed as the change in cell area/eccentricity at each time point compared to time point 0, as a function of mean concentration of Cytochalasin D that the cells were exposed to within this period of time (Fig. 6). The pooled data showed no significant trend in the change of cell area or cell eccentricity as a result of increase in Cytochalasin D concentration in time, which may suggest that inside the microfluidic device the cells were not exposed long enough to observe significant changes for a whole cell population, although a much faster response (as early as 5 min) was observed in the case of microplate cell culture. Nevertheless, the results showed a more pronounced data spreading in time, in particular for cell area, indicating that the cells indeed do respond to the treatment. However, it is plausible that in the presence of adherent cells, the stable gradient is established more slowly, than in the tests with the fluorescent dye only.

Taken together, the results of this study showed that the platform developed here is suitable for tracking the changes in cell morphology over time, upon exposure to a concentration gradient created inside the microfluidic device over a monolayer of MG-63 cells. Furthermore, gradients could be established in a shear-stress free regime, which involved the establishment of a diffusion based gradient between the source and the sink side channels. This set-up allowed for the study of single cells within each chamber while at the same time the heterogeneity within the whole cell population could be assessed. Moreover, gains in stability in the gradient formation from ealier timepoints are expected to improve accuracy of measurements and better sampling of cellular responses for the different concentration conditions. In addition, the platform here presented provides an insightful new contribution for low-concentration microfluidic screenings relying in cytometric measurements, proposing higher sampling by a series of culture chambers operated within a single seeding.

## Conclusions

In summary, a microfluidic glass-PDMS device consisting of five independent cell culture chambers, inside of which adherent cells can be exposed to a gradient of compounds in solution in a shear stress-free environment, was successfully developed. The modelling experiments confirmed that stable gradients can indeed be formed and the exposure of MG-63 osteosarcoma cells to Cytochalasin D showed a concentration-dependent effect on cell morphology in time. While Cytochalasin D was used here for the validation of the platform, the platform is suitable for screening other compounds of interest; and, since it contains two parallel fluid supply channels, even the overlapping gradients of two or more compounds can be created (bidirectional for this design), as was shown in several earlier studies, with analogous designs (Harink et al. 2015; Kilinc et al. 2016; Millet et al. 2010). The cell culture chamber design and the selected cell density allowed for image-based cytometry of cell morphology with a high resolution and the large cell culture area ensured a large sample size. The platform further allowed for several culture chambers to be run simultaneously, with single cell seeding. Platforms such as the one developed here represents an interesting and dynamic tool, for example for mechanotransduction studies, such as those focusing on the formation of focal adhesion complexes, ERK1/2 activation or MAPK signaling (Dupont et al. 2011; Aitken et al. 2006; Martineau and Gardiner 1985). Measuring cellular responses using microfluidic devices with diffusion based gradients may be advantageous over the use of static well plate based cultures, especially when it concerns determination of threshold concentrations for instance for screening purposes. For example, this platform could further be used for combinatorial studies into the effect of cell culture surface substrates and soluble gradients, currently of great interest within the study of topographies as instructive (bio)material cues (Sonam et al. 2016; Kundu et al. 2013; Yang et al. 2011).

## Electronic supplementary material

Supplementary Figure 1 – Schematic wireframe representation of a microfluidic unit containing the cell culture chamber, in the center, and two side channels through which the medium is perfused in the direction of the arrows. Scale numbers in millimeters. (PNG 132 kb)

Supplementary Figure 2 – Concentration gradient profiles in time in individual cell culture chambers (represented by the 5 lines) in the absence of cells. Intensity of CF™ 568 maleimide was measured in the red channel of the fluorescence microscope and quantified by Image J. (PNG 178 kb)

Supplementary Figure 3 – Measured area of MG-63 cells as a function of Cytochalasin D concentration at different time-points (0, 2, 15, 30, 60 and 90 min) in a control experiment, performed in 96 well microplate. (PNG 124 kb)
