# Supplementary figures and images for: Development of a shear stress-free microfluidic gradient generator capable of quantitatively analyzing single-cell morphology
Source: Biomed Microdevices. 2017 Sep 7;19(4):81. doi: 10.1007/s10544-017-0222-z (PMC5589786; doi:10.1007/s10544-017-0222-z)

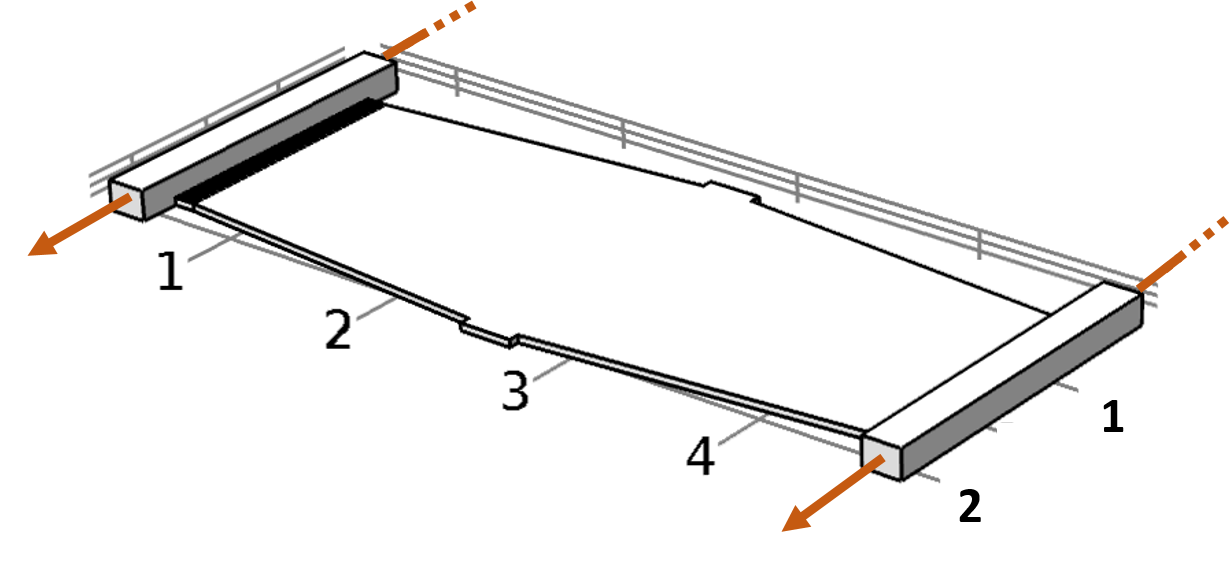

Supplement: Supplementary file 1 — – Schematic wireframe representation of a microfluidic unit containing the cell culture chamber, in the center, and two side channels through which the medium is perfused in the direction of the arrows. Scale numbers in millimeters. (PNG 132 kb) [file 10544_2017_222_MOESM1_ESM.png]

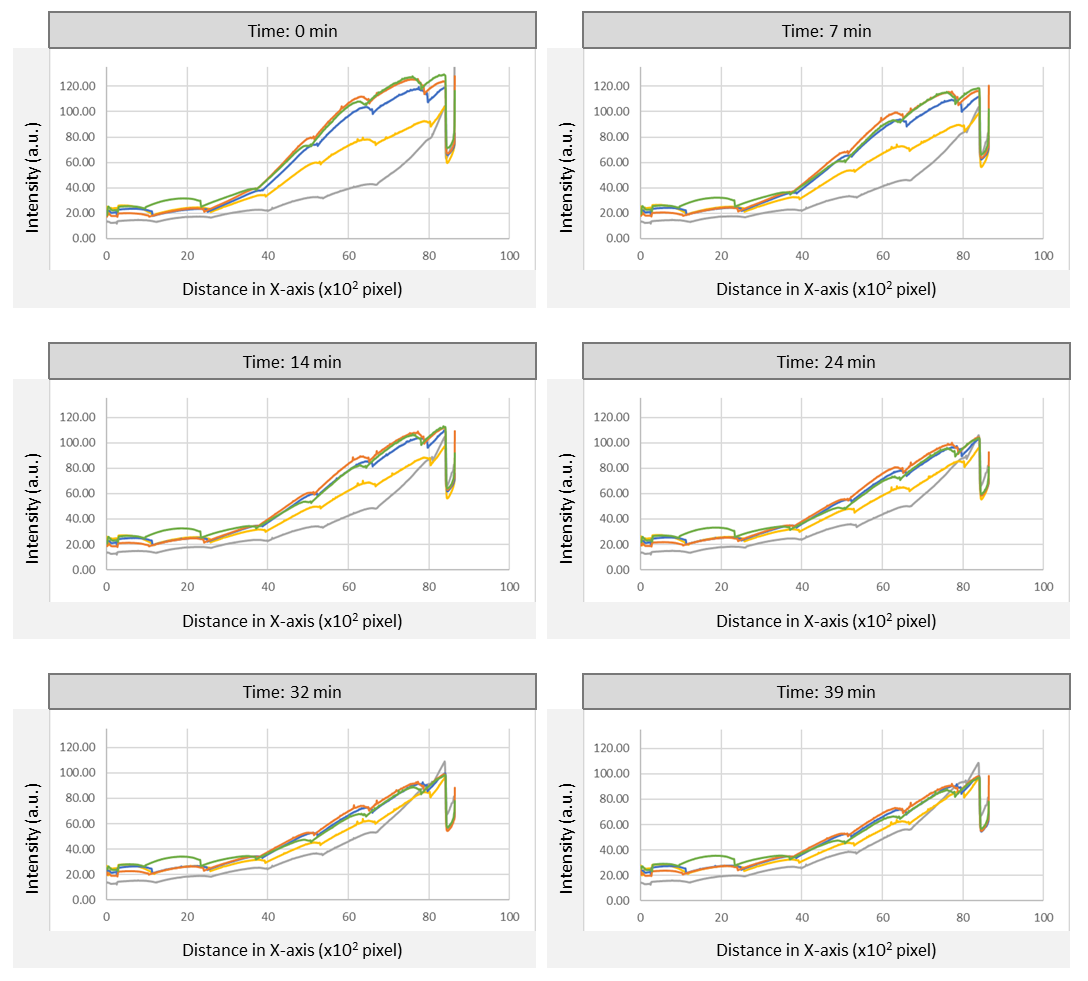

Supplement: Supplementary file 2 — – Concentration gradient profiles in time in individual cell culture chambers (represented by the 5 lines) in the absence of cells. Intensity of CF™ 568 maleimide was measured in the red channel of the fluorescence microscope and quantified by Image J. (PNG 178 kb) [file 10544_2017_222_MOESM2_ESM.png]

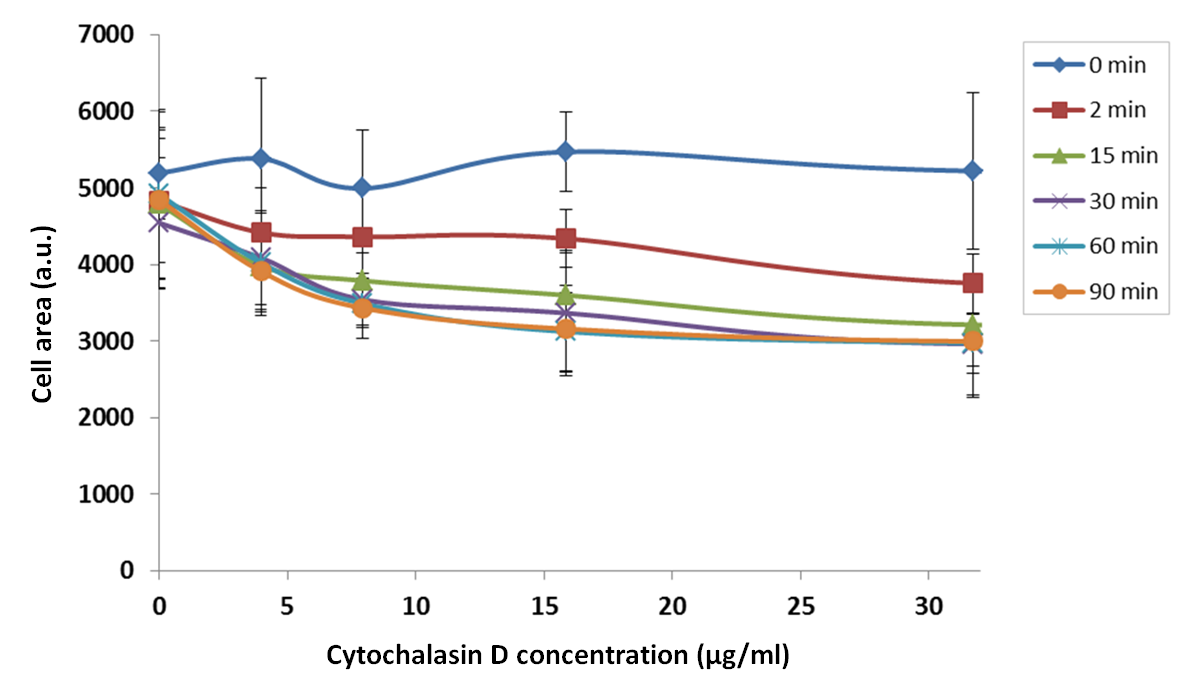

Supplement: Supplementary file 3 — – Measured area of MG-63 cells as a function of Cytochalasin D concentration at different time-points (0, 2, 15, 30, 60 and 90 min) in a control experiment, performed in 96 well microplate. (PNG 124 kb) [file 10544_2017_222_MOESM3_ESM.png]
